# Self-sorting heterodimeric coiled coil peptides with defined and tuneable self-assembly properties

**DOI:** 10.1038/srep14063

**Published:** 2015-09-15

**Authors:** Christopher Aronsson, Staffan Dånmark, Feng Zhou, Per Öberg, Karin Enander, Haibin Su, Daniel Aili

**Affiliations:** 1 Division of Molecular Physics, Department of Physics, Chemistry and Biology, Linköping University, 581 83 Linköping, Sweden; 2 School of Materials Science and Engineering, Nanyang Technological University, Singapore 639798; 3 Vehicular Systems, Department of Electrical Engineering, Linköping University, 581 83 Linköping, Sweden

## Abstract

Coiled coils with defined assembly properties and dissociation constants are highly attractive components in synthetic biology and for fabrication of peptide-based hybrid nanomaterials and nanostructures. Complex assemblies based on multiple different peptides typically require orthogonal peptides obtained by negative design. Negative design does not necessarily exclude formation of undesired species and may eventually compromise the stability of the desired coiled coils. This work describe a set of four promiscuous 28-residue de novo designed peptides that heterodimerize and fold into parallel coiled coils. The peptides are non-orthogonal and can form four different heterodimers albeit with large differences in affinities. The peptides display dissociation constants for dimerization spanning from the micromolar to the picomolar range. The significant differences in affinities for dimerization make the peptides prone to thermodynamic social self-sorting as shown by thermal unfolding and fluorescence experiments, and confirmed by simulations. The peptides self-sort with high fidelity to form the two coiled coils with the highest and lowest affinities for heterodimerization. The possibility to exploit self-sorting of mutually complementary peptides could hence be a viable approach to guide the assembly of higher order architectures and a powerful strategy for fabrication of dynamic and tuneable nanostructured materials.

The coiled coil is one of the most abundant protein structural motifs found in nature. It is especially common in fibrous proteins such as myosin, keratin and fibrinogen^1^. Computational analysis has established that about 3% of all amino acids in the known genomes are involved in coiled coil structures^2^. As reported by Crick, Pauling and Corey in the 1950’s, coiled coils can be described as two or more α-helices that intertwine, creating rope-like structures^1^,^3^. The amino acid sequence of coiled coils typically show a heptad repetition of hydrophobic (**h**) and polar (**p**) residues in a **hpphppp** pattern, commonly denoted as (*abcdefg*)_n_ where n is the number of repeats and *a*-*g* show the relative positions of the amino acids in a helical wheel diagram (Fig. 1). The hydrophobic effect is the main driving force for association and folding of coiled coils. Hydrophobic residues at *a* and *d* positions pack together in a “knobs-into-hole” fashion to minimize their interaction with water molecules^1^. The stability of coiled coils is also affected by the presence of charged residues at *e* and *g* positions. Oppositely charged residues stabilize inter-helical arrangements whereas like charges lead to repulsion and destabilization. The heptad repeat pattern makes coiled coils fairly straightforward to de novo design and design rules have been extensively investigated, as summarized in several review articles^4^,^5^,^6^,^7^,^8^.

Heterodimeric coiled coils with tuneable and well-defined assembly properties are of great interest as model systems to mimic and study biomolecular interactions involved in for example vesicle fusion^9^, and cell-signalling events^10^. Coiled coils have also been exploited as components in a range of supramolecular materials, including peptide-polymer hybrid hydrogels^11^,^12^, peptide fibers^13^, and peptide-nanoparticle hybrids^14^. Assembly of complex peptide nanostructures require simultaneous use of multiple peptides that displays a varying degree of complementarity. Recent beautiful examples include the assembly of a peptide tetrahedron by Jerala *et al.*^15^, based on six different orthogonal coiled coil forming peptide pairs, as well as a number of complex but structurally well-defined nanostructured assemblies described by Woolfson *et al.*^16^.

To be able to control the self-assembly process and stability of peptide-based supramolecular nanostructures, it is critical to have tuneable and quantified affinities for oligomerization^17^. Several different strategies have been developed to obtain coiled coils with defined oligomeric states, helix orientations and affinities for association^18^,^19^,^20^. The length of a peptide greatly affects its potential to interact with other peptides. Increasing the number of heptads typically increases the affinity for oligomerization. Recently, Thomas *et al.* described a set of heterodimeric coiled coils with tuneable and quantified dissociation constants in the micromolar to sub-nanomolar range obtained by varying the numbers of heptad repeats^21^. Although highly successful for tuning the interaction strength the use of peptides of different length in the same coiled coil motif may result in problems with fringing and unspecific interactions caused by the overhanging ends.

In order to reduce unwanted interactions and to prevent formation of undesired folded species, negative design elements are typically introduced. Commonly, polar or charged amino acids at *a* or *d* or a pinpointed charge interaction at specific *e* and *g* positions, are introduced^22^,^23^,^24^. Efforts to reduce formation of undesired oligomers and folds may however result in an overall decrease in stability of the intended structures. In contrast, by positive design, desired species are favoured by introducing stabilizing elements. The unwanted species can still form but the equilibrium is shifted towards the thermodynamically most stable structures. Example of positive design elements for coiled coils are to include more hydrophobic residues at *a* and *d*^25^.

Here, we investigate the possibility to form specific heterodimers with high fidelity from mixtures of complementary peptides based on large differences in affinities. We describe a set of four peptides capable of forming parallel heterodimeric coiled coils with dissociation constants spanning from low micromolar to the picomolar range. The affinity for dimerization was modulated using the well-known differences in packing efficiencies of the two β-branched amino acids valine and isoleucine in the hydrophobic core and the difference in α-helical propensities of serine and alanine^25^. Using this strategy we obtained four rather similar 28-residue peptides that can be combined into four different parallel heterodimeric coiled coils. The conformations and stabilities were investigated using circular dichroism (CD) spectroscopy and molecular dynamics (MD) simulations. When all peptides were combined, the large differences in dissociation constants promoted social self-sorting and assembly of only two of the four possible coiled coils. The possibility to exploit self-sorting of mutually complementary peptides to guide the assembly of higher order architectures is an interesting alternative to the use of orthogonal peptides that further enables fabrication of dynamic and tuneable materials and nanostructures.

## Results and Discussion

### Peptide design

Four different 28 amino acid residue coiled coil forming peptides (EV, EI, KV, KI) were developed (Table 1) following established coiled coil design rules^26^. The sequences were based upon peptides designed by Hodges *et al.*^20^,^25^, and Woolfson *et al.*^21^, but with some modifications in order to obtain two sets of complementary peptides with large differences in affinities for dimerization. In short, the peptides were designed to heterodimerize at neutral pH by adding complementarily charged residues at *e* and *g* using glutamic acid (Glu, E) for EV and EI and lysine (Lys, K) for KV and KI. The hydrophobic face of the helices has leucine (Leu, L) at position *d* and a β-branched amino acid at position *a*. Valine (Val,V) was used at position *a* for EV and KV peptides whereas isoleucine (Ile, I) was included at this position for EI and KI. These amino acids were chosen in order to obtain a wide span of affinities for dimerization for the four possible combinations of coiled coil heterodimers (EVKV, EVKI, EIKV and EIKI). An asparagine (Asn, N) residue was incorporated at *a* in the third heptad in all peptides to promote formation of dimeric structures with parallel orientation^21^,^26^. The asymmetric positioning of the polar Asn in the hydrophobic core will also favour assembly of in-register structures because of favourable Asn-Asn′ pairing between two peptides. The helix promoting amino acid alanine (Ala, A) was used at position *b* in all peptides and at position *c* in the EI and KI peptides. In the EV and KV peptides, position *c* was instead occupied by serine (Ser, S) that has slightly lower α-helix propensity compared to Ala. To balance the net charge of the peptides and to increase water solubility Glu residues were included at position *f* for the KV and KI. For EV and EI, Lys was used instead. In the third heptad a tryptophan (Trp, W) was incorporated at *f* as a chromophore.

### Peptide characterization

**Figure 2:**
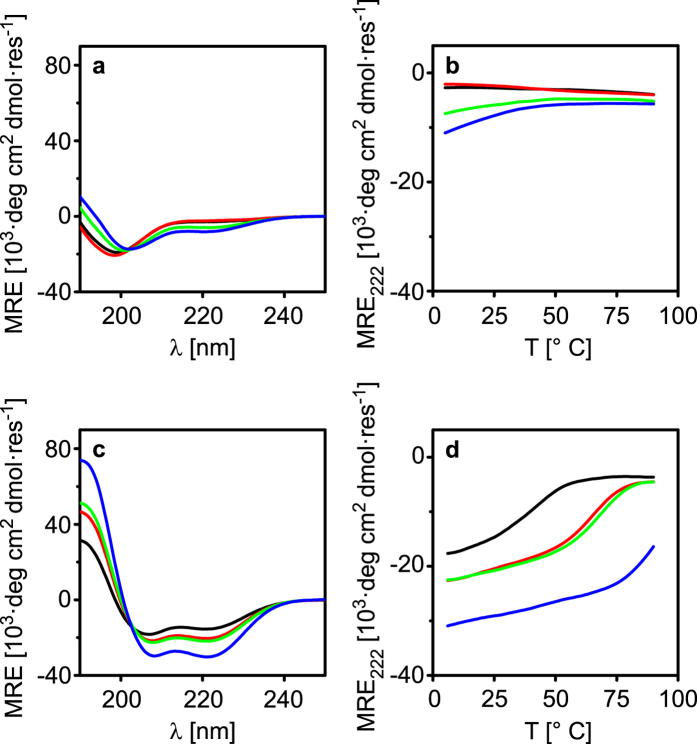
CD spectra of (a) monomers at 20 °C (b) monomer thermal denaturation (c) coiled coils at 20 °C (d) coiled coils thermal denaturation. Key: (**a**,**b**) EV = black; KV = red; EI = green; KI = blue. (**c**,**d**) EVKV = black; EVKI = red; EIKV = green; EIKI = blue.

The peptides were synthesized on solid support using Fmoc chemistry and their secondary structure and folding properties were characterized using CD spectroscopy. CD spectra of the individual peptides showed that the peptides were mainly random coils at neutral pH (Fig. 2a). This indicates that the charge repulsion provided by residues at *e* and *g* was sufficient to prevent homodimerization and folding. A slight shift of the negative band from 198 nm in the spectra for EV and KV to 201–202 nm for EI and KI indicate that the latter two may exhibit a small amount of secondary structure. This is presumably because Ile is slightly more hydrophobic compared to Val but also provides a better packing of the hydrophobic core^20^. It should also be noted that the negative band shift is slightly more pronounced for KI than for EI. This can be explained by the longer and more flexible side chain in Lys compared to Glu. The ε–amino groups in neighbouring Lys residues can be positioned further apart as compared to the γ–carboxyls in two adjacent Glu residues. A lower charge repulsion between KI monomers than between EI monomers is thus expected. A similar tendency has been observed for Fos-Jun heterodimers that also can form Jun-Jun homodimers^27^.

Thermal denaturation experiments showed that only a moderate increase in temperature abolished all tendencies of folding for EI and KI (Fig. 2b, Supplementary Table S1). This clearly indicates that the interactions between peptides of the same kind are very dynamic and unstable with melting temperatures (T_m_) lower than 20 °C. By changing the pH closer to the pI of the individual peptides it is possible to trigger homooligomerization and folding of the peptides (Supplementary Figure S1, Table S2). The EI and KI homooligomers showed impressive thermal stabilities with T_m_ > 85 °C, whereas EV had a T_m_ of 67 °C. KV did only show minor tendencies to associate with T_m_ of <30 °C.

Mixing the different monomers at pH 7 generated CD spectra with two minima at 208 and 222 nm for all four possible heterodimer combinations, indicating formation of α-helical structures (Fig. 2c). The ratio of the two minima ([Θ]_222_/[Θ]_208_) provides an estimate of the extent of coiled coil formation^28^. A ratio >1 indicates well-defined coiled coils whereas lower values indicate a less defined coiled coil structure. EVKV had the lowest ratio (0.85) whereas EIKI showed the highest ratio (1.01). EVKI and EIKV did also display relatively high [Θ]_222_/[ Θ]_208_ ratios, 0.94 and 0.97 respectively. Addition of 50 vol% of 2,2,2-Trifluoroethanol (TFE), a compound that disrupts hydrophobic cores of coiled coils while maintaining the α-helical conformation of individual peptides, resulted in a decrease in the [Θ]_222_/[Θ]_208_ ratio for all dimers (Supplementary Figure S2). For EVKV this decrease was fairly small, from 0.85 to 0.83. For EVKI and EIKV the ratio decreased to 0.84 while the ratio for EIKI decreased to 0.85. All dimers, except EVKV, hence showed a significant change in the [Θ]_222_/[Θ]_208_ ratio upon addition of TFE. This indicates that these peptide combinations do form defined and stable coiled coils.

Thermal denaturation experiments showed a distinct difference in stability of the different heterodimers (Fig. 2d). EVKV, EVKI and EIKV all demonstrated true monophasic sigmoidal melting curves, indicating discrete and cooperatively folded structures. EIKI did also show this trend in the measureable temperature range (<90 °C) but the peptide did not unfold completely even at the highest temperatures. T_m_ ranged from 37 to 87 °C for the different pairs where EVKV and EIKI showed the lowest and the highest T_m_ values, respectively (Table 2). An isobestic point at 203 nm was also seen for all heterodimers further indicating that there indeed was a discrete unfolding from α-helices to random coil (Supplementary Figure S3)^29^.

The method of continuous variation was utilized to examine the oligomerization state of the coiled coils. The molar ratio of each monomer was varied and the ellipticity at [Θ]_222_ was measured for all ratios (Supplementary Figure S4). This confirmed that all four peptide combinations primarily associate in a 1:1 ratio. Formation of dimers was verified using several predictive analysing tools, including Logicoil^30^, RFCoil^31^, and PrOCoil^32^.

**Figure 3:**
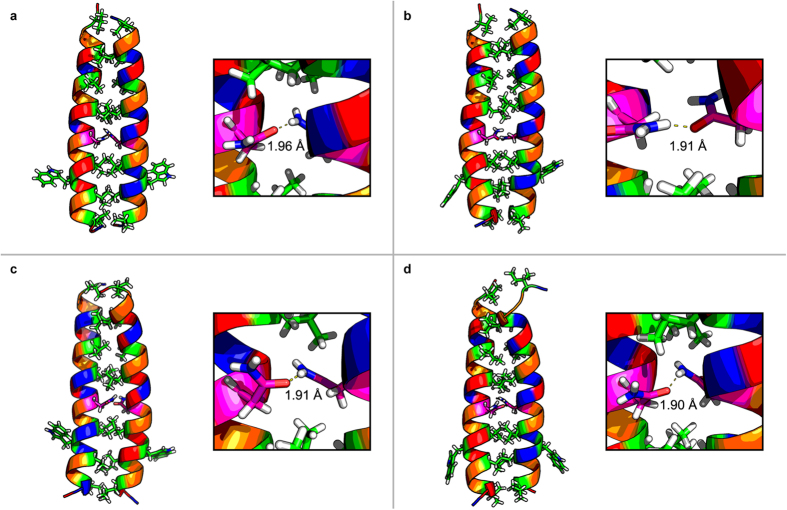
Molecular dynamics simulations of (a) EVKV, (b) EVKI, (c) EIKV and (d) EIKI, showing formation of parallel heterodimeric coiled coils. EIKI showed the tightest packing of the residues in the hydrophobic core of all four coiled coils, as indicated by the shorter hydrogen bond length for Asn-Asn′. Amino acids are coloured according to Lesk Scheme^45^.

Formation of transient or less stable oligomers can however not be fully excluded but MD simulations further supported formation of stable parallel heterodimers for all peptides pairs. Within 40 ns all peptide pairs had formed parallel heterodimers (Fig. 3). No coiled coil assembly was seen within this time frame when the simulations were started with the peptides aligned in an anti-parallel orientation. Moreover, Asn pairing via hydrogen bonding could also be observed, supporting that Asn will guide the peptides to dimerize in a parallel orientation and in-register. The Asn-Asn′ hydrogen bond distance was 1.96 Å in EVKV and 1.90 Å in EIKI. For EVKI and EIKV this distance was 1.91 Å in both peptide pairs. This agrees well with the CD data since a shorter hydrogen bond distance indicates a more tightly packed and stable dimer structure.

The dissociation constants (K_d_) for all heterodimers were estimated from thermal denaturation data recorded at different peptide monomer concentrations. T_m_ values were estimated and related to K_d_ (Supplementary Figure S5)^33^. The lowest K_d_ value at 20 °C was obtained for EIKI (K_d_ < 0.1 nM), whereas EVKV showed significantly lower affinity for heterodimerization (K_d_ = 1.4 μM). EVKI and EIKV had K_d_ values in the nanomolar range, 85 and 72 nM, respectively. By changing from Val-Val′ at *d* in the hydrophobic core to Val-Ile′ it is thus possible to increase the affinity for dimerization by a factor of ~20. In contrast, using Ile-Ile′ pairing the affinity for heterodimerization was increased by more than four orders of magnitude as compared to Val-Val′.

### Temperature dependent self-sorting

**Figure 4:**
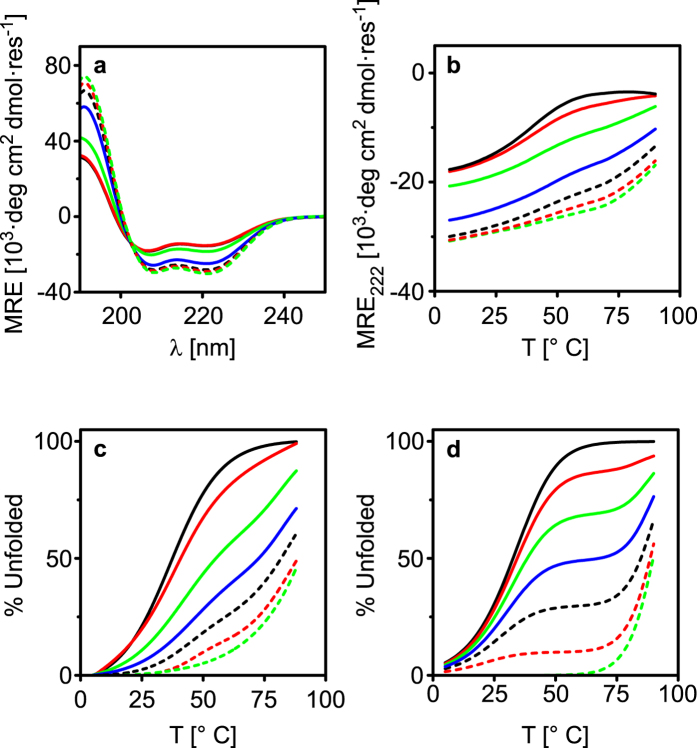
(**a**) CD spectra at 20 °C for different χ_%_ [EI:KI]. (**b**) Thermal denaturation curves for different χ_%_ [EI:KI]. (**c**) Normalized thermal denaturation curves for different χ_%_ [EI:KI] showing the % of unfolding and (**d**) % of unfolding obtained from simulations for different χ_%_ [EI:KI] and temperatures. Key: χ_0%_ [EI:KI] = black, solid; χ_10%_ [EI:KI] = red, solid; χ_30%_ [EI:KI] = green, solid; χ_50%_ [EI:KI] = blue, solid; χ_70%_ [EI:KI] = black, dash; χ_90%_ [EI:KI] = red, dash; χ_100%_ [EI:KI] = green, dash.

The peptides are obviously promiscuous since no negative design elements have been introduced to prevent formation of any of the four possible heterodimers. Moreover, all heterodimers display reasonably low dissociation constants, albeit spanning over a wide range. Because of the large differences in affinities for heterodimerization, the peptides are expected to undergo thermodynamic self-sorting when all of them are mixed. To investigate this hypothesis, EV, KV, EI and KI were mixed at different molar ratios and the thermal stability of the ensemble was characterized using CD spectroscopy. The total peptide concentration was kept constant at 50 μM and the ratios for EV:KV and EI:KI was kept at 1:1, respectively. Instead the relative mole percent (χ_%_) of [EI:KI] compared to [EV:KV] was varied from 0 to 100%. Since the different dimers have defined melting temperatures the number of transitions in the thermal denaturation curve of the mixtures reflects the number of different dimers present. Increasing the χ_%_ of [EI:KI] at 20 °C indeed increased the overall helicity and [Θ]_222_/[Θ]_208_ ratio (Fig. 4a, Supplementary Table S3). This is expected because of the relative higher concentration of EIKI dimers formed. Increasing the χ_%_ of [EI:KI] also resulted in a higher overall thermal stability of the ensemble (Fig. 4b, Supplementary Table S3).

The degree of unfolding for different ratios was estimated by normalizing the denaturation curves using a two-state unfolding model (Fig. 4c). In the span from χ_10%_ to χ_90%_, two major visible thermal transitions were obtained, one in the lower temperature span (<50 °C) and one in the higher (>80 °C), corresponding to the unfolding of EVKV and EIKI heterodimers, respectively. From the derivative of the thermal denaturation curves the presence of only two thermal unfolding transitions is clearly seen (Supplementary Figure S6). Computer simulations based on the K_d_ values of the dimers at different temperatures showed the same trend as well with only two thermal unfolding transitions (Fig. 4d).

**Figure 5:**
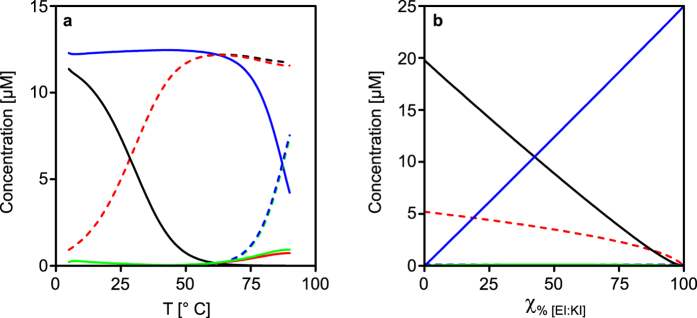
(**a**) Simulations of χ_50%_ [EI:KI] as a function of temperature. At 20 °C the monomers self-sort into predominantly EVKV and EIKI and only 2% forms EVKI or EIKV dimers. (**b**) Simulated equilibrium concentrations of monomers and heterodimers when varying χ_%_ [EI:KI] at 20 °C. The relative amount of EVKV and EIKI will be roughly the same at χ_42%_ [EI:KI]. For other χ_%_ [EI:KI], see supporting information. Key: EV = black, dash; KV = red, Dash; EI = green, dash; KI = blue, dash; EVKV = black, solid; EVKI = red, solid; EIKV = green, solid; EIKI = blue, solid.

From computer simulations of all dimer combinations (Fig. 5a and Supplementary Figure S8) it is evident that the peptides predominantly assemble into EVKV and EIKI. A certain amount of EVKI and EIKV forms as well but at χ_50%_ and 20 °C to a very small extent, less than 2% of all dimers. Increasing the temperature above T_m_ for EVKV leads to an increase in the concentration of free EV and KV monomers. The concentration of EIKI stays fairly constant at temperatures below 60 °C because of the high thermal stability of this heterodimer. The low concentration of free EI and KI monomers thus prevents formation of EVKI and EIKV. Above 75 °C EIKI will start to dissociate to a larger extent. The resulting increase in concentration of free EI and KI will thus slightly increase the probability for formation of EVKI and EIKV heterodimers. At the same time the corresponding K_d_ values is relatively high under these conditions (>0.2 μM at 75 °C) and the concentration of these heterodimers will hence be low. Since EIKI has about two orders of magnitude lower K_d_ at this temperature, more than 92% of all dimers will be in the form of EIKI. By keeping the temperature fixed and instead varying the ratio of [EI:KI] to [EV:KV] it would in theory be possible to tune the relative equilibrium dimer concentrations (Fig. 5b). For example, at about χ_42%_ of [EI:KI] at 20 °C there will be equal concentrations of EVKV and EIKI heterodimers in a peptide mixture.

**Figure 6:**
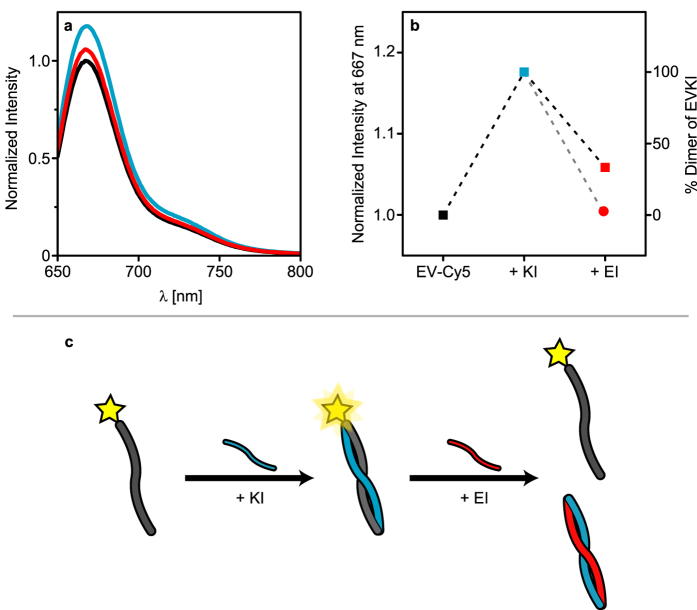
(**a**) Fluorescence spectra and (**b**) change in normalized intensity at 667 nm for EV-Cy5 upon addition of EI and KI. Addition of the complimentary peptide KI, thus forming EVKI dimers, results in an increase in fluorescence intensity. Subsequent addition of EI reduces the fluorescence intensity due to formation of EIKI dimers, leaving free EV-Cy5. The experimental data correlate well with simulations. (**c**) Schematic representation of the experiment. Key: **(a)** EV-Cy5 = black, solid; +KI = blue, solid; 3) +EI = red, solid. (**b**) EV-Cy5 = black, square; +KI = blue, square; +EI = red, square; (simulation) +EI = red, circle.

To further experimentally verify that the peptides undergo thermodynamic self-sorting we modified EV at the N-terminus with a fluorescent dye (Cy5). Although not considered very environmentally sensitive, cyanine dyes tethered to the terminus of single-stranded DNA display significant changes in fluorescence quantum yield, emission lifetime, and fluorescence anisotropy decay upon hybridization with a complementary DNA strand^34^,^35^. Addition of KI (5 μM) to EV-Cy5 (5 μM) resulted in increased emission intensity due to formation of (EV-Cy5)KI heterodimer (Fig. 6a,b). Subsequent addition of EI (5 μM) resulted in a drop in emission intensity, close to the original intensity of the monomeric EV-Cy5. Since KI has a significantly higher affinity for EI than for EV, the addition of EI leads to a transition from (EV-Cy5) KI to EIKI heterodimers and EV-Cy5 monomers as illustrated in Fig. 6c. This change in binding partner was also supported by simulations (Fig. 6b). The smaller amount of monomeric EV-Cy5 seen in the experiment after addition of EI is presumably an effect of the kinetics involved in the exchange of partners in the heterodimer. The simulations were run until equilibrium whereas the experimental data was acquired after 30 minutes incubation and the exchange was likely not fully completed within this time frame. Addition of EI prior to KI, but under otherwise identical conditions, instead resulted in a slight decrease in fluorescence intensity similar to the photo-bleaching of the dye upon acquisition of repeated spectra (Supplementary Figure S7).

## Conclusions

By using only four different de novo designed peptides it is possible to obtain four different parallel coiled coil heterodimers. Their dissociation constants span from micromolar to picomolar at room temperature and because of the large difference in affinities, the peptides undergo thermodynamic social self-sorting when combined. Predominately two out of four possible heterodimers were formed, as shown by thermal denaturation curves of different peptide combinations, along with fluorescence data and simulations. In previous work, self-sorting has primarily been imposed by introducing negative design elements to generate fully orthogonal peptide sequences. In contrast, the peptides described here are not designed to exclude certain combinations of heterodimers based on unfavourable interactions and all four heterodimers can thus form. The possibility to form defined heterodimers with high fidelity from mixtures of non-orthogonal peptides can facilitate efforts to realize defined complex polypeptide nanostructures. The distinct self-sorting properties of the peptide heterodimers presented here will thus be a valuable addition to the rich toolbox of coiled coil peptides available in the literature. Furthermore, the possibility to tune the strength of association by different hydrophobic core packing and the fact that they undergo social self-sorting makes these peptides highly attractive supramolecular building blocks in dynamic peptide-based nanomaterials. The wide range of affinities opts for possibilities to tune materials properties with high precision, which can be used for e.g. fabrication of peptide-based hydrogels with defined viscoelastic properties for tissue engineering, 3D cell culturing and drug delivery, and for tuneable self-assembly of peptide-nanoparticle hybrids for sensing applications.

## Methods

### General

Fmoc-protected amino acids were purchased from Iris Biotech GmbH. HCTU and DIPEA were acquired from Protein Technologies, Inc. DMF and acetonitrile were obtained from VWR International. All other chemicals were acquired from Sigma Aldrich. All measurements were carried out in phosphate buffer (PB; mono- and disodium phosphate, total concentration 10 mM) and adjusted to pH 7 using phosphoric acid or sodium hydroxide. Peptide concentrations were determined by UV absorbance (ε_280_(Trp) = 5690 M^−1^ cm^−1^). All measurements were at 50 μM total peptide concentration unless otherwise noticed. All samples were allowed to equilibrate for 30 min after mixing before measurement.

### Peptide Synthesis and purification

The peptides were obtained by solid phase peptide synthesis on a Symphony Quartet from Protein Technologies, Inc. As solid phase H-Rink-Amide ChemMatrix resin (0.47 mmol of NH_2_/g of resin) was used on a 100 μM scale. Synthesis was performed using a standard Fmoc protocol with a 6-fold molar excess of amino acids and HCTU as coupling agent. N-terminal acetylation was carried out in acetic anhydride/DMF (1:1). For peptides used in fluorometric measurements Cy5-NHS was used instead of acetic anhydride at this step. The peptides were cleaved from the resin with mixtures of triflouroacetic acid (TFA)/triisopropylsilane/water (95:2.5:2.5, 90 min). Peptides were filtered from the resin and the TFA/peptide mixture was concentrated to approximately 1 ml under a flow of nitrogen. The crude peptides were then precipitated in −20 °C cold diethyl ether and isolated by centrifugation and subsequently thoroughly washed. The isolated product were dissolved in 1:1 acetonitrile/water and lyophilized to a dry powder.

Purification was done by reverse phase HPLC using a preparative Kromatek HiQ-Sil C18HS column (250 by 21.1 mm) on a Dionex Ultimate 3000 LC system from Thermo Scientific. Depending on sequence different buffers were used. Peptides with a positive net charge (K-peptides) were purified by running linear gradients of water and acetonitrile (Buffer A, 90% water, 9.9% acetonitrile and 0.1% TFA; Buffer B, 10% water, 89.9% acetonitrile and 0.1% TFA). Peptides with a negative net charge (E-peptides) were purified by running linear gradients of triethylammonium acetate buffer (TEAA-buffer, 20 mM triethylamine, 20 mM acetic acid, adjusted to pH 9) and acetonitrile (Buffer C, 90% TEAA-buffer, 10% acetonitrile; Buffer D, 10% TEAA-buffer, 90% acetonitrile). Linear gradients were from 10% to 40% buffer B or D during 8.5 min with a constant flowrate of 20 ml/min. All peptides were monitored and collected based on UV-signal at 229 nm and 274 nm. Final purity was controlled by reverse phase HPLC and the mass identity was confirmed by MALDI-ToF mass spectrometry with α-cyano-4-hydroxycinnamic acid as matrix.

### Circular Dichrosim (CD) Spectroscopy

CD spectra and thermal denaturation curves were recorded using a Chirascan^TM^ spectropolarimeter from Applied Photophysics with a TC 125 temperature controller from Quantum Northwest. CD spectra were measured between 190 and 250 nm at 20 °C in quartz cuvettes with a step size of 0.5 nm. Thermal curves were measured by heating from 5 to 90 °C at 1 °C intervals with 0.2 °C accuracy and at a rate of roughly 50 °C per hour. The CD signal at 222 nm was recorded for each temperature. These curves were fitted to either a mono- or biphasic sigmoidal function depending on whether one or two transitions was observed, and corresponding melting temperatures were estimated from these. Each CD experiment was run in triplicates and data was averaged and converted to mean residue ellipticity (MRE, [Θ]). Normalization of ratio thermal denaturation curves was done by using equation (1).


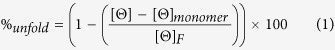


that is a modified version of Litowski and Hodges^36^. [Θ] is the measured MRE at 222 nm and [Θ]_monomer_ is the estimated MRE at 222 nm in a mixture of monomers if no interaction occur between them. These value are estimated from thermal denaturation experiments of the monomers. [Θ]_F_ is the MRE at maximum folding, for simplification estimated as the value of ([Θ] − [Θ]_monomer_) at 5 °C. When [Θ] = [Θ]_monomer_ it is assumed that complete unfolding has occurred.

### Molecular dynamics simulations

Simulations were performed using the GROMACS package^37^. The overall temperature of the water and peptides was kept constant, coupling each group of molecules independently at 300 K with a V-rescale thermostat^38^. The pressure was coupled to a Parrinello-Rahman^39^,^40^ barostat at 1 atm separately in every dimension. The temperature and pressure time constants of the coupling were 0.1 and 2 ps, respectively. Integration of equations of motion was performed by using a leap frog algorithm with a time step of 2 fs. Periodic boundary conditions were implemented in all systems. A cut-off of 1 nm was implemented for the Lennard–Jones and the direct space part of the Ewald sum for Coulombic interactions. The Fourier space part of the Ewald splitting was computed by using the particle-mesh-Ewald method^41^, with a grid length of 0.16 nm on the side and a cubic spline interpolation. TIP3P water model^42^ was used and the peptide parameters were from the CHARM force field^43^,^44^.

### Self-sorting simulations

Simulations were carried out using MATLAB R2014b (The MathWorks, Inc.). Details, including scripts, can be found in supplementary information.

## Additional Information

**How to cite this article**: Aronsson, C. *et al.* Self-sorting heterodimeric coiled coil peptides with defined and tuneable self-assembly properties. *Sci. Rep.* **5**, 14063; doi: 10.1038/srep14063 (2015).

## Supplementary Material

Supplementary Information

## Figures and Tables

**Figure 1:**
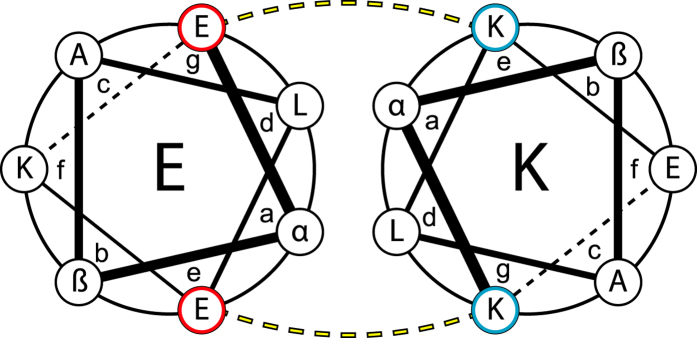
A helical wheel diagram showing the coiled coil heptad repeat for a heterodimer. Residues at *a* and *d* creates a hydrophobic core while *e* and *g* residues favours heterodimerization through formation of stabilizing salt bridges. For peptides used in this work, α = Val or Ile (3^rd^ heptad Asn), β = Ala or Ser.

**Table 1:** Peptide sequences with corresponding heptad register.

	*gabcdef*	*gabcdef*	*gabcdef*	*gabcdef*
EV	EVSALEK	EVSALEK	ENSALEW	EVSALEK
KV	KVSALKE	KVSALKE	KNSALKW	KVSALKE
EI	EIAALEK	EIAALEK	ENAALEW	EIAALEK
KI	KIAALKE	KIAALKE	KNAALKW	KIAALKE

**Table 2:** CD characterization data for the different heterodimers.

	[Θ]_222_ at 20 °C [10^3^∙deg cm^2^ dmol∙res^−1^]	[Θ]_222_/[Θ]_208_ at 20 °C		T_m_ [°C]	K_d_ [M] at 20 °C
		PB	TFE		
EVKV	−15.4	0.85	0.83	37.4 ± 0.1	1.4 ± 1.5 × 10^−6^
EVKI	−20.4	0.94	0.84	61.6 ± 0.4	8.5 ± 7.1 × 10^−8^
EIKV	−21.8	0.97	0.84	63.7 ± 0.5	7.2 ± 4.7 × 10^−8^
EIKI	−30.1	1.01	0.85	86.7 ± 0.04	<1.0 × 10^−10^ (0.43 ± 4.9 × 10^−16^)*

*Calculated value
