# Supplementary material for: Self-sorting heterodimeric coiled coil peptides with defined and tuneable self-assembly properties
Source: Sci Rep. 2015 Sep 15;5:14063. doi: 10.1038/srep14063 (PMC4570195; doi:10.1038/srep14063)
Supplement: Supplementary Information [file srep14063-s1.pdf]

Self-sorting heterodimeric coiled coil peptides with defined and tuneable self-assembly properties

Christopher Aronsson,[1] Staffan Dånmark,[1] Feng Zhou,[2] Per Öberg,[3,4] Karin Enander,[1] Haibin Su,[2] & Daniel Aili.[1]

[1]Division of Molecular Physics, Department of Physics, Chemistry and Biology, Linköping University, 581 83 Linköping, Sweden.

[2]School of Materials Science and Engineering, Nanyang Technological University, Singapore 639798.

[3]Vehicular Systems, Department of Electrical Engineering, Linköping University, 581 83 Linköping, Sweden.

[4]Current address: Wolfram MathCore, Teknikringen 1F, 583 30 Linköping, Sweden.

Correspondence and requests for materials should be addressed to D.A. (email: daniel.aili@liu.se).

Supplementary Information

Table of contents

1. Additional tables S2
2. pH-dependence S3
3. TFE experiment S5
4. Thermal curves S5
5. The method of continuous variation S6
6. K_d measurements S7
7. Derivative of thermal denaturation curves for different □$_{\%\ [EI:KI]}$ S8
8. Fluorescence Data of EV-Cy5 and addition of EI S8
9. Simulations S9
10. Main scripts for MATLAB simulations S10
11. Peptide identity and purity S12
12. References S14

1. Additional tables

	$[\Theta]_{222}$ at 20° C [10^3·deg cm^2 dmol·res^{-1}]	$[\Theta]_{222}/[\Theta]_{208}$ at 20° C	T_m [° C]
EV	- 3.96	0.36	< 5
KV	- 2.40	0.29	< 5
EI	- 5.94	0.56	< 20
KI	- 8.02	0.63	< 20

Table S1. Mean residue ellipicities at 222 nm, fraction 222/208 and melting temperatures for each peptide monomer.

	$[\Theta]_{222}$ at 20° C [10^3·deg cm^2 dmol·res^{-1}]	$[\Theta]_{222}/[\Theta]_{208}$ at 20° C	T_m [° C]
EV (pH 2)	- 21.9	0.94	66.6 ± 0.5
KV (pH 12)	- 8.24	0.68	29.4 ± 0.4
EI (pH 2)	- 32.7	1.04	> 90
KI (pH 12)	- 27.2	1.01	86.3 ± 0.3

Table S2. Mean residue ellipicities at 222 nm, fraction 222/208 and melting temperatures for each peptide monomer at specified pH.

$\chi_{\%\ [EI:KI]}$	$[\Theta]_{222}$ at 20° C [10^3·deg cm^2 dmol·res^{-1}]	$[\Theta]_{222}/[\Theta]_{208}$ at 20° C	$T_{m\ mean}$ [° C]
0	- 15.4	0.85	37.4 ± 0.1
10	- 15.2	0.85	41.3 ± 0.6
30	- 18.4	0.90	53.9 ± 0.03
50	- 24.9	0.96	69.5 ± 0.05
70	- 28.0	0.99	78.6 ± 0.03
90	- 29.0	1.01	84.6 ± 0.04
100	- 30.1	1.01	86.7 ± 0.04

Table S3. Mean residue ellipicities at 222 nm, fraction 222/208 nm and peptide ensemble mean melting temperatures for each $\chi_{\%}$ of [EI:KI] in peptide mixtures.

2. pH-dependence

By varying the pH it is possible to reduce destabilizing charge-charge repulsion, resulting in peptide homooligomerization and folding. At pH < 3 Glu at *e* and *g* in EV/EI will be fully protonated and at pH > 11 Lys at *e* and *g* in KV/KI will be deprotonated (Figure S1A). Figure S1B shows that α-helical structures are formed to different extent for the different peptides. Thermal denaturation experiments shows that T_m ranges from 30° C to >90° C for the different structures (Figure S1C, Table S2).

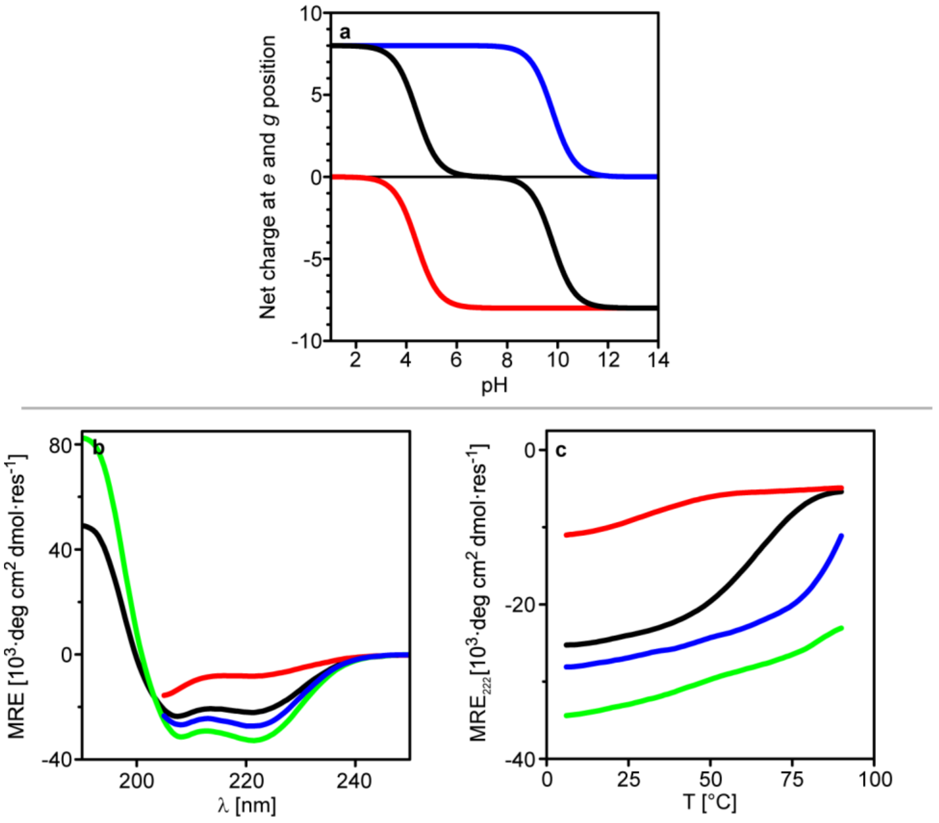

Figure S1. (a) Calculated net charge at *e* and *g* position for homo- and heterodimers. When the net charge is close to zero, peptides will be able dimerize. **(b)** CD spectra of monomers at different pH. Due to increased noise at higher pH for wavelengths < 200 nm, only data between 205 - 250 nm is displayed for the K-peptides. **(c)** Thermal denaturation curves for monomers at different pH. Key: **(a)** Mixture of E-peptide and K-peptide = black; E-peptide = red; K-peptide = blue. **(b,c)** EV at pH 2 = black; KV at pH 12 = red; EI at pH 2 = green; KI at pH 12 = blue.

The difference in stability is probably due to the type of supramolecular interaction that can be formed between *e* and *g* residues. At pH 12 Lys ε-amino groups will be fully deprotonated and hence be able to oligomerize KV/KI by hydrogen bond formation.[1] One of the slightly positive hydrogen atoms will be able to form a stabilizing bond with one electronegative nitrogen in the Lys - Lys´ interactions between two monomers. Due to the directionality of hydrogen bonding only one of two possible hydrogen bond per Lys - Lys´ will be able to form, hence leaving a slightly positive hydrogen atom unbound.[2] This leads to a lower total bonding energy compared to corresponding

E/K-heterodimer at pH 7 where Glu - Lys´ form salt bridges. At pH 2 Glu's γ-carboxyl will be protonated and be able to oligomerize EV/EI peptides. In this case two hydrogen bonds will be able to form per Glu - Glu´ interaction since two slightly positive hydrogen and two electronegative ketone groups are available.[1] This gives a higher total bonding energy than corresponding supramolecular interaction between *e* and *g* at pH 7 and 12 respectively.

3. TFE experiment

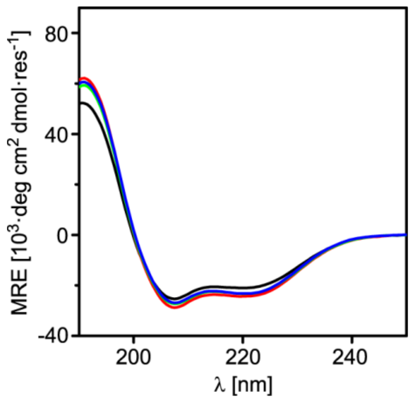

Figure S2. CD spectra for the different dimers after addition of 50 vol% trifluoroethanol. Key: black = EVKV; red =EVKI; green = EIKV; blue = EIKI.

4. Thermal curves

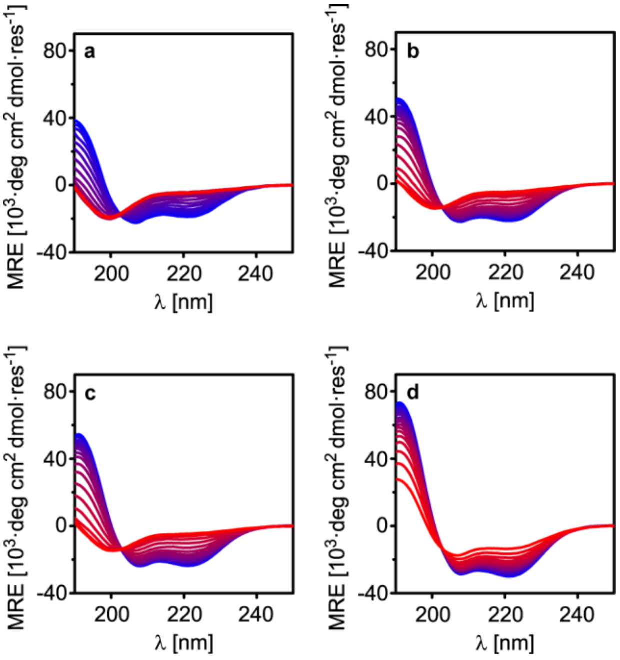

Figure S3. CD spectra of **a)** EVKV **b)** EVKI **c)** EIKV **d)** EIKI at different temperatures. By increasing the temperature an isobestic point at 203 nm can be observed indicating that we have a two-state transition, from α-helix to random coil. Key: “blue to red” - gradient is 5 to 90° C in steps of 5° C.

5. The method of continuous variation

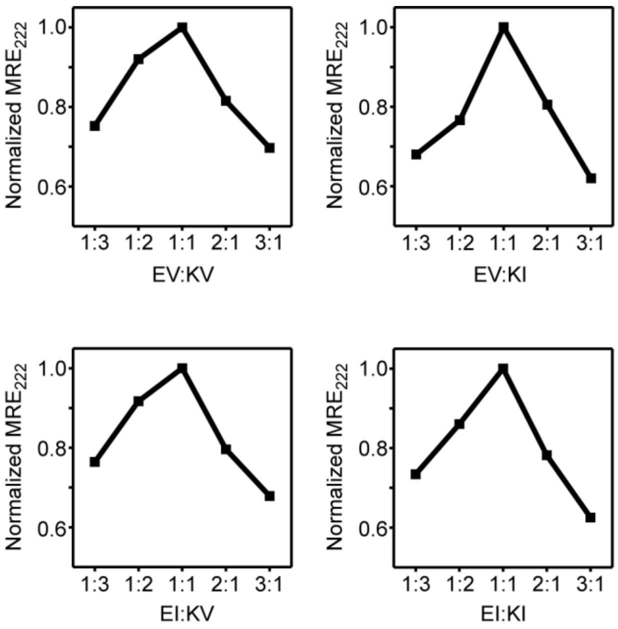

Figure S4. Jobs plot of all possible combinations of peptides. The lowest $[\Theta]_{222}$ was found to be at 1:1 ratio for all combinations. The graphs shows $[\Theta]_{222}$ of each ratio normalized to the lowest $[\Theta]_{222}$ measured for each combination.

6. K_d measurements

The dissociation constants (K_d) were determined using the method described by Marky and Breslauer[3]. In short, thermal denaturation curves were measured over a range of concentrations (200, 150, 100, 50 and 25 µM) by circular dichroism spectroscopy at 222 nm. For each measurement it was assumed that there is a transition from dimers to monomers (1). By using a model describing equilibrium of non-self-complementary association and defining the melting temperature T_m as where 50 % of all peptides are folded, (2) can be used (C_T = total peptide concentration).

$$A_1 + A_2 \leftrightarrow A_1A_2 \qquad \textbf{(1)}$$

$$K_a^{T_m} = \frac{[A_1A_2]}{[A_1][A_2]} = \frac{1/2}{(C_T/2)(1-1/2)^2} = \frac{4}{C_T} = \frac{1}{K_d^{T_m}} \qquad \textbf{(2)}$$

For any process in equilibrium $\Delta G^\circ = -RT\,ln\left(\frac{1}{K_d}\right)$ and $\Delta G^\circ = \Delta H^\circ - T\Delta S^\circ$. By rearranging we get

$$-RT\,ln\left(\frac{1}{K_d}\right) = \Delta H^\circ - T\Delta S^\circ \qquad \textbf{(3)}$$

By using (2) and (3) we can get an expression of K_d at T_m in terms of C_T. By dividing this with $T_m\Delta H^\circ$ and rearranging we get

$$\frac{1}{T_m} = \frac{R}{\Delta H^\circ}\,ln(C_T) + \frac{(\Delta S^\circ - R\,ln(4))}{\Delta H^\circ} \qquad \textbf{(4)}$$

By using this relationship and plotting $1/T_m$ versus $ln(C_T)$ it is possible to linearly extrapolate and determine a peptide concentration that would give a T_m of interest or to estimate the K_d at any temperature.

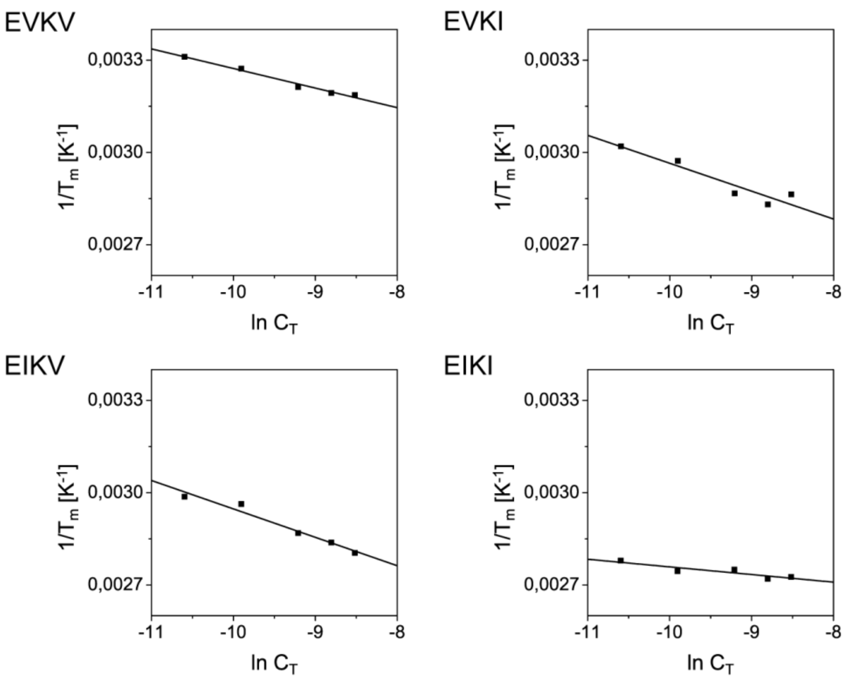

Figure S5. K_d measurements for each heterodimer. The reciprocal T_m was plotted against the natural logarithm of the total peptide monomer concentration (25, 50, 100, 150 and 200 µM). From this, K_d was estimated.

7. Derivative of thermal denaturation curves for different $\chi_{\%\,[EI:KI]}$

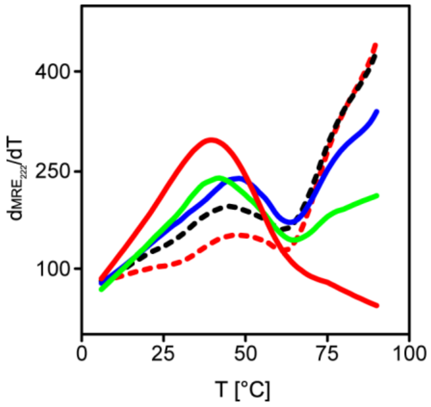

Figure S6. Derivative of thermal denaturation curves for the span $\chi_{10\%}$ to $\chi_{90\%}$ in figure 4C. Key: $\chi_{10\%}$ [EI:KI] = red, solid; $\chi_{30\%}$ [EI:KI] = green, solid; $\chi_{50\%}$ [EI:KI] = blue, solid; $\chi_{70\%}$ [EI:KI] = black, dash; $\chi_{90\%}$ [EI:KI] = red, dash.

8. Fluorescence Data of EV-Cy5 and addition of EI

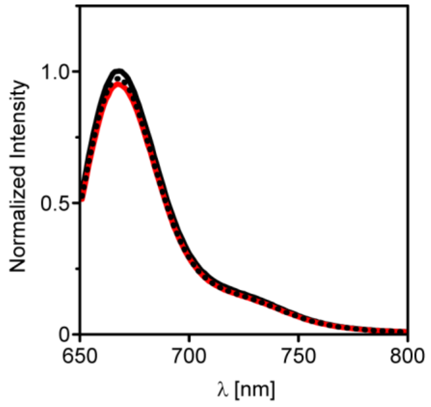

Figure S7. Fluorescence data of EV-Cy5 with addition of EI and compared to average photo-bleaching of the dye. Key: EV-Cy5 = black, solid; EV-Cy5 with added EI = red, solid; Average photo-bleach of EV-Cy5 = black, dot.

9. Simulations

For each ratio and temperature the amount of monomers versus dimers were calculated. K_d values at temperatures between 5 to 90° C were estimated using the method by Marky and Breslauer, described in section 6 (K_d measurements). For EIKI dimer, the lowest possible K_d values were fixed at 1 pM until a higher value was estimated (≥ 40° C). The simulations were run until convergence and no further changes were observed.

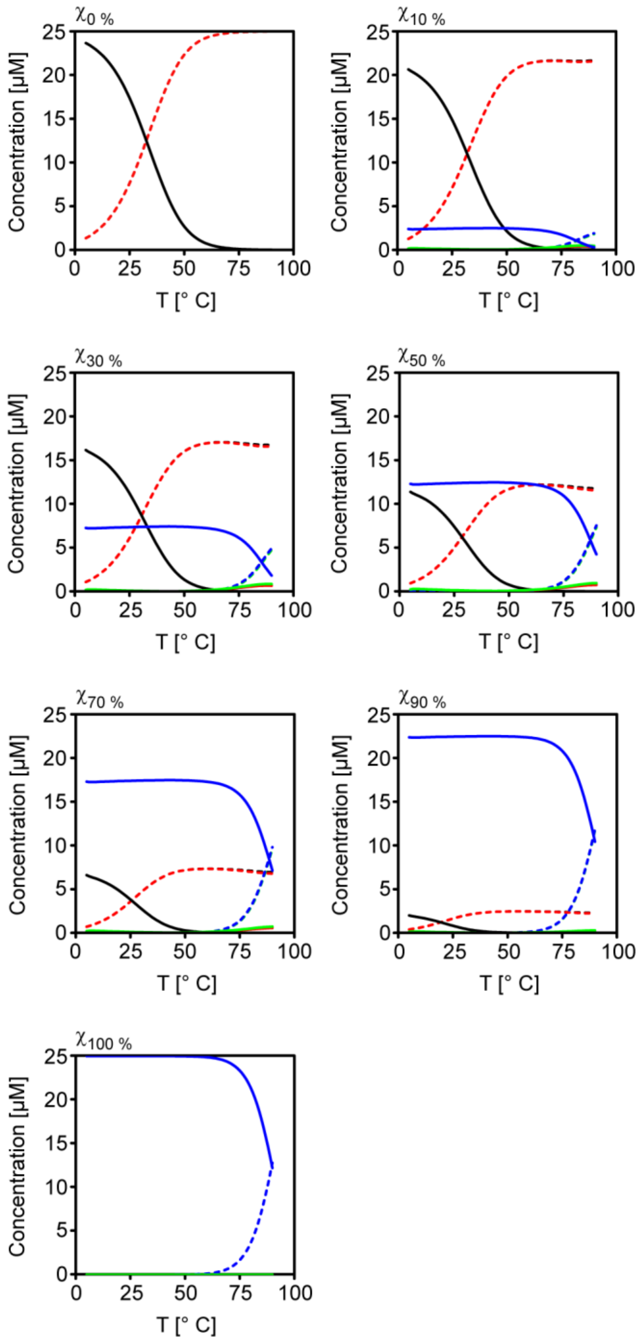

Figure S8. Simulation data of peptide monomer and dimer concentrations at different molar ratios ($\chi_{\%}$) of [EI:KI] as a function of temperature. The monomers self-sorts into predominantly EVKV and EIKI dimers. Key: EV = black, dash; KV = red, Dash; EI = green, dash; KI = blue, dash; EVKV = black, solid; EVKI = red, solid; EIKV = green, solid; EIKI = blue, solid.

10. Main scripts for MATLAB simulations

Calculation for each ratio

```
%% Start at 5 degree Celsius, using the function "calculation"

    eqConc{1} = calculation(data.EIKI(1),data.EIKV(1),data.EVKI(1),data.EVKV(1),xEV,xKI,xKV,xEI,0,0,0,0,1E-
9);
    eqConc{1}.deg = data.deg(1);

%% Loop for 6 to 90 degree Celsius, using the function "calculation"

for ii = 2:length(data.deg)

    eqConc = calculation (data.EIKI(ii),data.EIKV(ii),data.EVKI(ii),data.EVKV(ii),eqConc{ii-1}.xEV,eqConc{ii-
1}.xKI,eqConc{ii-1}.xKV,eqConc{ii-1}.xEI,eqConc{ii-1}.xEIKV,eqConc{ii-1}.xEVKI,eqConc{ii-
1}.xEIKI,eqConc{ii-1}.xEVKV,1E-9);
    eqConc.deg = data.deg(ii);
end

theFields = fieldnames(eqConc{1});

for kk = 1:length(data.deg)
for ii = 1:length(theFields)
   eqConcNew.(theFields)(kk) = eqConc.(theFields) ;
end
end
```

Calculation for each temperature

```
function x_out = calculation(k1,k2,k3,k4,xEV,xKI,xKV,xEI,xEIKV,xEVKI,xEIKI,xEVKV,numloops)

kconv = 10^(-9); %% Convergence factor

for ii = 1:numloops

xEIKI_n = xEIKI - kconv*(xEIKI - xEI*xKI / k1);
xEIKV_n = xEIKV - kconv*(xEIKV - xEI*xKV / k2);
xEVKI_n = xEVKI - kconv*(xEVKI - xEV*xKI / k3);
xEVKV_n = xEVKV - kconv*(xEVKV - xEV*xKV / k4);

xEI_n = xEI - (xEIKI_n-xEIKI) - (xEIKV_n - xEIKV);
xKI_n = xKI - (xEIKI_n-xEIKI) - (xEVKI_n - xEVKI);
xEV_n = xEV - (xEVKI_n-xEVKI) - (xEVKV_n - xEVKV);
xKV_n = xKV - (xEVKV_n-xEVKV) - (xEIKV_n - xEIKV);

xEI = xEI_n;
xEV = xEV_n;
xKI = xKI_n;
xKV = xKV_n;

xEIKI = xEIKI_n;
xEIKV = xEIKV_n;
xEVKI = xEVKI_n;
xEVKV = xEVKV_n;

end


x_out.xEI = xEI;
x_out.xEV = xEV;
x_out.xKI = xKI;
x_out.xKV = xKV;

x_out.xEIKI = xEIKI;
x_out.xEVKI = xEVKI;
x_out.xEVKV = xEVKV;
x_out.xEIKV = xEIKV;
```

11. Peptide identity and purity

EV

Sequence: Acetyl – EVSALEK EVSALEK ENSALEW EVSALEK – NH_2

MALDI ToF-MS ($mass_{calc}$ = 3159.4 g/mol)

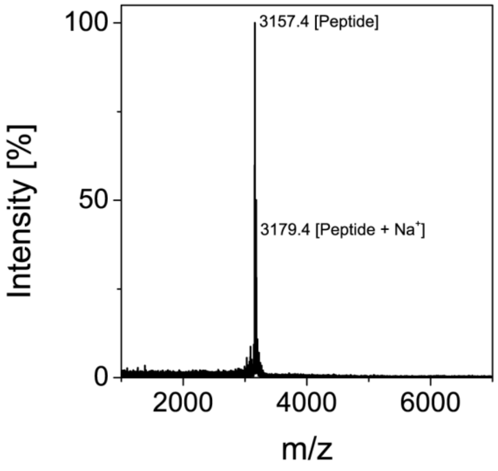

HPLC (gradient 10-80 % D in 20 min, 20 ml/min, UV = 229 nm)

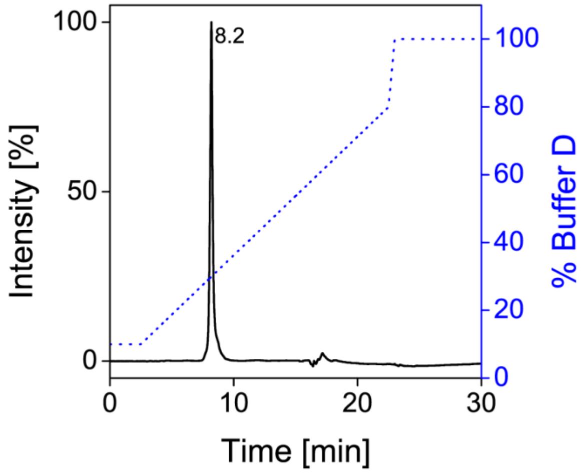

KV

Sequence: Acetyl – KVSALKE KVSALKE KNSALKW KVSALKE – NH_2

MALDI ToF-MS ($mass_{calc}$ = 3154.8 g/mol)

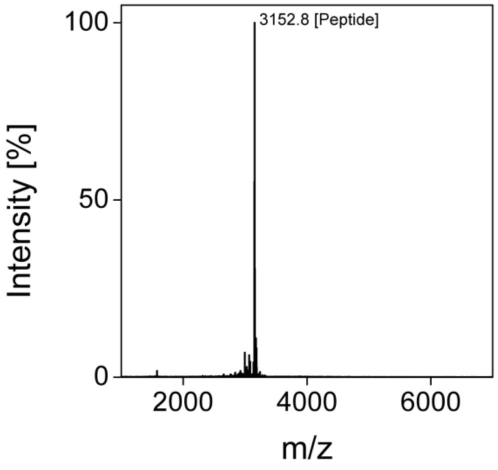

HPLC (gradient 10-80 % B in 20 min, 20 ml/min, UV = 229 nm)

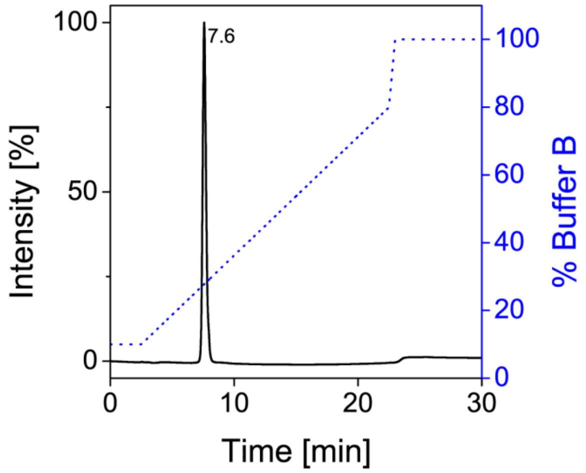

EI

Sequence: Acetyl – EIAALEK EIAALEK ENAALEW EIAALEK – NH_2

MALDI ToF-MS ($mass_{calc}$ = 3137.6 g/mol)

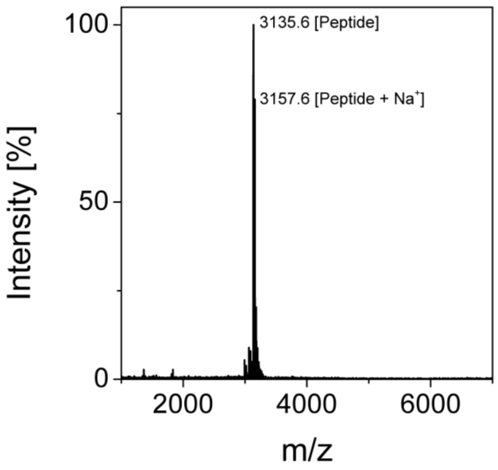

HPLC (gradient 10-80 % D in 20 min, 20 ml/min, UV = 229 nm)

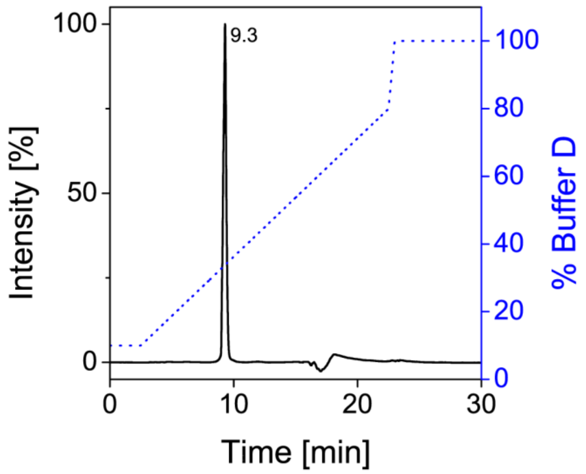

KI

Sequence: Acetyl – KIAALKE KIAALKE KNAALKW KIAALKE – NH_2

MALDI ToF-MS ($mass_{calc}$ = 3132.9 g/mol)

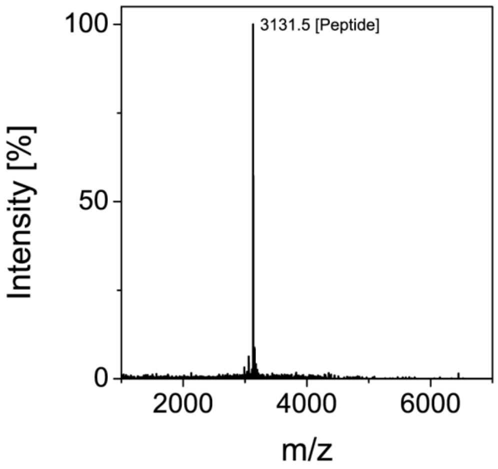

HPLC (gradient 10-80 % B in 20 min, 20 ml/min, UV = 229 nm)

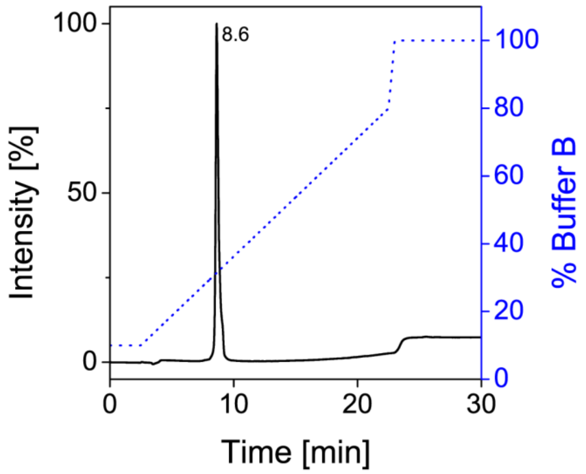

12. References

1 Solomons, T. W. G. & Fryhle, C. B. *Organic chemistry*. 9th edn, (Wiley, 2008).

2 Gilli, G. & Gilli, P. *The nature of the hydrogen bond. Outline of a comprehensive hydrogen bond theory*. (OUP Oxford, 2009).

3 Marky, L. A. & Breslauer, K. J. *Calculating thermodynamic data for transitions of any molecularity from equilibrium melting curves.* Biopolymers 26, 1601-1620, (1987).
